# Nonlinear relationship between Hemoglobin-to-Age Ratio and all-cause mortality in patients with septic shock: A retrospective cohort study in the MIMIC-IV database

**DOI:** 10.1371/journal.pone.0313937

**Published:** 2024-12-06

**Authors:** Chao Yang, Yimin Xue, Zhebin You, Tingfeng Huang, Xiaofang He, Xinda Jiang, Jianmin Huang, Yu Chen, Xiao-Fen Zhou

**Affiliations:** 1 Fuzhou University Affiliated Provincial Hospital, Fuzhou, Fujian, People’s Republic of China; 2 Shengli Clinical Medical College of Fujian Medical University, Fuzhou, Fujian, People’s Republic of China; 3 The Fourth Department of Critical Care Medicine, Fujian Provincial Hospital, Fuzhou, Fujian, People’s Republic of China; 4 Department of Geriatric Medicine, Shengli Clinical Medical College of Fujian Medical University, Fuzhou, People’s Republic of China; 5 Fujian Key Laboratory of Geriatrics Diseases, Fujian Provincial Center for Geriatrics, Fujian Provincial Hospital, Fuzhou, Fujian, People’s Republic of China; 6 Department of Stomatology, Fujian Provincial Hospital, Fuzhou, Fujian, People’s Republic of China; 7 Digestive Endoscopy Center, Fujian Provincial Hospital, Fuzhou, Fujian, People’s Republic of China; 8 Department of Anesthesiology, Fujian Provincial Hospital, Fuzhou, Fujian, People’s Republic of China; 9 Fujian Provincial Key Laboratory of Emergency Medicine, Fuzhou, Fujian, People’s Republic of China; 10 Fujian Provincial Key Laboratory of Critical Care Medicine, Fuzhou, Fujian, People’s Republic of China; Pescara General Hospital, ITALY

## Abstract

**Background:**

Previous studies have shown that both age and hemoglobin are closely associated with the prognosis of septic shock. A recent study found that hemoglobin may change with age. Hemoglobin-to-Age Ratio (HAR) takes both age and hemoglobin into consideration as essential factors. So far, the effect of HAR on the prognosis of septic shock is still unclear. This research aimed to investigate the association between the HAR and the prognosis of patients with septic shock.

**Methods:**

Cox proportional hazards regression analysis, restricted cubic spline, Kaplan-Meier survivor analysis and stratified interaction analysis were used to elucidate the relationship between the HAR and prognosis of patients with septic shock.

**Results:**

There is a nonlinear association between the HAR and mortality within 28 days after intensive care unit admission. When the HAR was lower than 0.13, mortality within 28 days after ICU admission decreased significantly as the HAR increased. When the HAR was higher than 0.13, the HAR was not a protective factor for mortality within 28 days after ICU admission. In patients with septic shock, the HAR was more effective in reducing the risk of death in patients with atrial fibrillation than in patients without atrial fibrillation.

**Conclusion:**

There is a nonlinear association between the HAR and mortality within 28 days after intensive care unit admission. When the HAR was at a low level, mortality within 28 days after ICU admission decreased significantly as the HAR increased. When the HAR was at high levels, the HAR might not be a protective factor for mortality within 28 days after ICU admission. In patients with septic shock, the HAR was more effective in reducing the risk of death in patients with atrial fibrillation than in patients without atrial fibrillation.

## 1 Introduction

Sepsis is a life-threatening organ dysfunction caused by a dysregulated host response to infection [1]. Septic shock (SS) is the most serious complication of sepsis [2]. Epidemiologic findings have shown the mortality of SS remains as high as 30% [3,4]. Previous studies have shown that both age and hemoglobin (Hb) concentration are closely associated with the prognosis of SS [5,6]. A recent study found that hemoglobin concentration may change with age [7]. There are limitations in applying Hb alone to evaluate the prognosis of SS. The Hemoglobin-to-Age Ratio (HAR) takes age and Hb into consideration as essential factors and corrects for the effect of age on hemoglobin. So far, no studies have been reported on the association between the HAR and the prognosis of SS. Our research assessed the role of the HAR in predicting all-cause mortality in patients with SS and provided additional methods for evaluating the prognosis of SS.

## 2 Materials and methods

### 2.1 Study population

This study is a retrospective observational study. The data of the study population were all obtained from the MIMIC-IV (version 2.2) database. The MIMIC-IV database consists of high-quality medical records of patients admitted to the Intensive care unit (ICU) of Beth Israel Deaconess Medical Center [8]. One author (Chao Yang) complied with the requirements for access to the database and was responsible for the data extraction.

Inclusion criteria: (1) Patients diagnosed with SS according to the 9th and 10th revisions of the International Classification of Diseases (ICD); (2) Patients requiring ICU admission.

**Fig 1:**
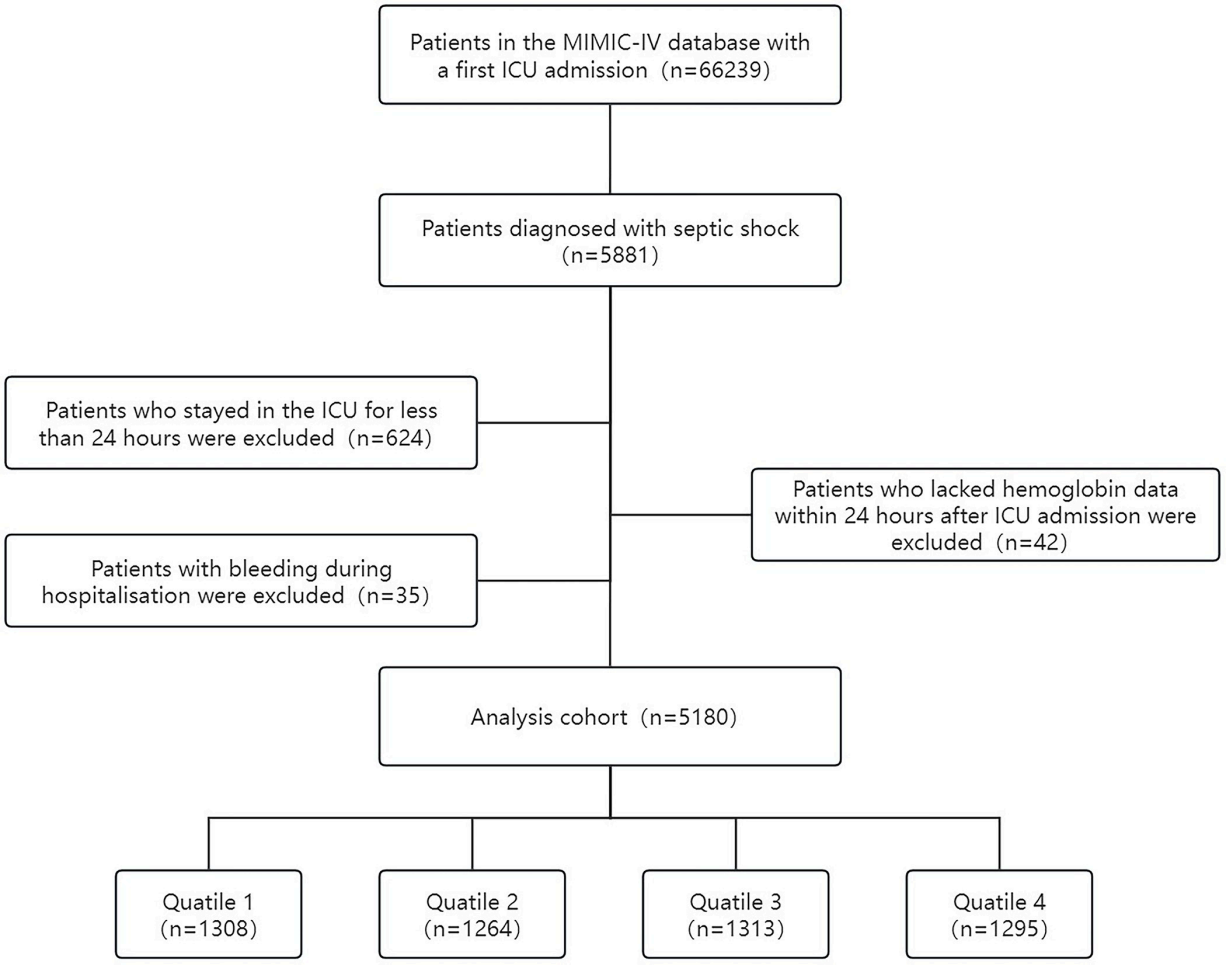
Flow of included patients through the trial.

Exclusion criteria: (1) Patients whose age at the time of admission to the ICU was less than 18 years old; (2) Patients whose length of stay in the ICU was less than 24 hours; (3) Patients who lacked data of Hb within 24 hours after ICU admission; (4) For patients with multiple ICU admissions, we only extracted data from their first ICU admission. (5) Patients with bleeding complications during hospitalization. Finally, a total of 5180 patients were enrolled in this study (S1 Raw data). All patients were grouped into four groups based on the quartiles of the HAR, where the first quartile of the HAR was regarded as the reference group (Fig 1).

### 2.2 Data collection

Potential variables were divided into six main categories: (1) Demographics, including age, gender, and race. (2) Comorbidities, including asthma, diabetes, acute pancreatitis (AP), atrial fibrillation (AF), acute myocardial infarction (AMI), and acute kidney injury (AKI). (3) Laboratory tests within 24 hours after first admission to the ICU, including white blood cells (WBC), Hb, platelets, serum creatinine (Scr), blood urea nitrogen (BUN), International Normalized Ratio (INR), serum potassium, serum sodium, serum chloride and serum total calcium. (4) Disease severity scores within 24 hours after first admission to the ICU, including the Sequential Organ Failure Assessment (SOFA) score, the Simplified Acute Physiology Score II (SAPS II). (5) Total erythrocyte transfusion (TET) within 28 days after admission to the ICU. Variables were excluded if they had more than 20% missing data. Age and hemoglobin were used to calculate the HAR. The formula was HAR = hemoglobin (g/dl)/ Age(year). Follow-up started at the time of first ICU admission and ended on the 28th day after the first ICU admission or when the endpoint events occurred.

### 2.3 Endpoint events

The endpoint event of this study was death within 28 days after ICU admission.

### 2.4 Statistical analysis

Continuous variables were presented as mean ± standard deviation or median (interquartile range). Categorical variables were presented as proportions. The Kolmogorov-Smirnov test was used to assess the normality of continuous variables. Continuous variables that were normally distributed were analyzed by t-test or ANOVA; Continuous variables that were not normally distributed were analyzed by the Kruskal-Wallis test. Qualitative variables were analyzed by chi-square test. The effects of the different levels of the HAR on endpoint events were evaluated using the Kaplan-Meier survivor analysis (log-rank test).

**Fig 2:**
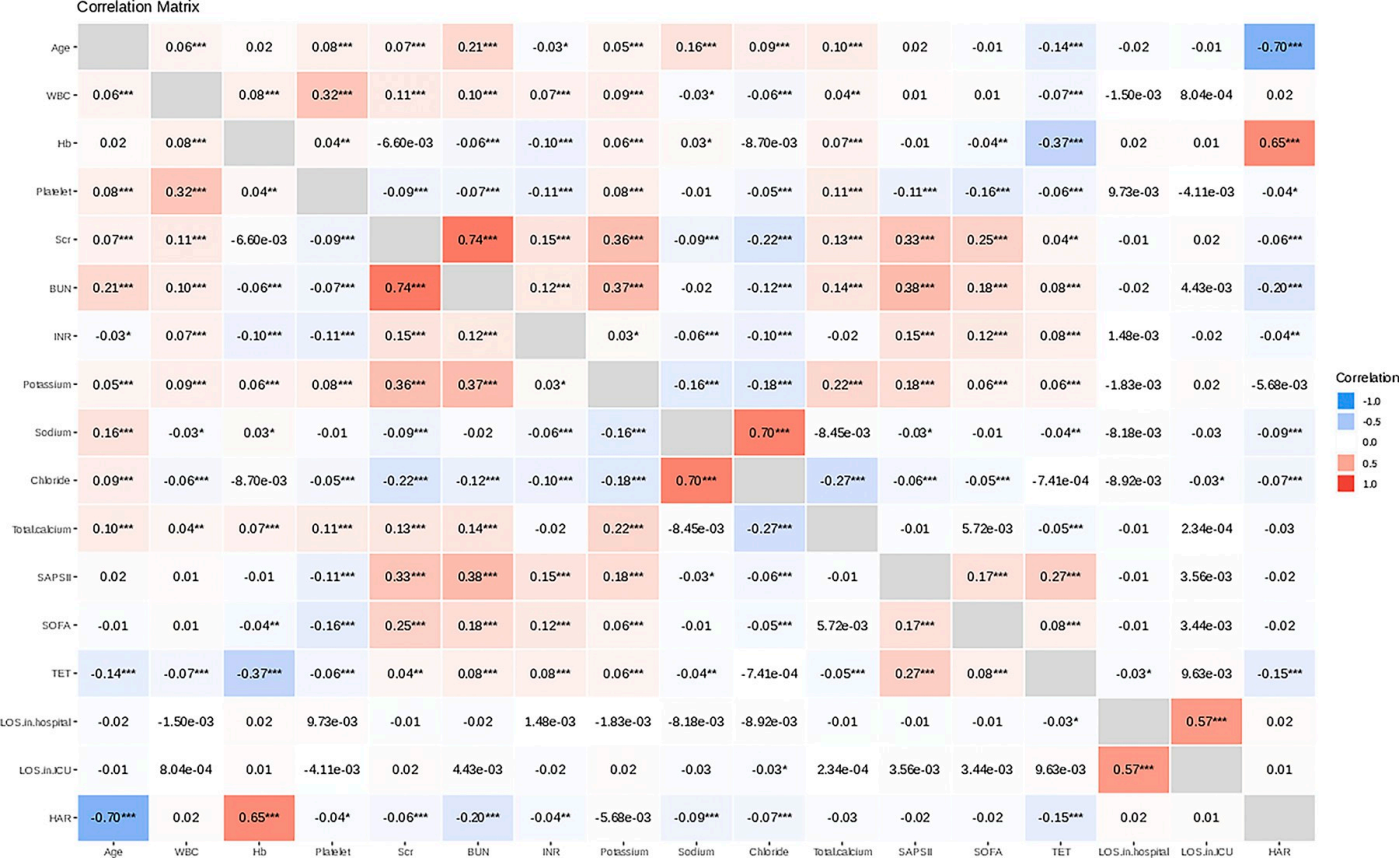
Correlation matrix.

Hazard ratios (HR) and 95% confidence intervals (CI) for the risk of endpoint events that were associated with elevated HAR were estimated using Cox proportional hazards models with adjustment for covariates. Criteria for adjustment covariates: (1) In the univariate analysis, the P-value of the regression coefficient of the covariate on the mortality within 28 days after ICU admission is less than 0.1. (2) Inclusion of covariate in the basic model or exclusion of covariate from the complete model affects regression coefficients of the HAR by more than 10%. (3) We performed correlation analyses for continuous variables and constructed correlation matrix to exclude multicollinearity between variables (Fig 2). We applied scatterplots to illustrate the relationships between some variables (see S1 to S9 Figs). Finally, the covariates that were selected included sex, AF, AMI, AKI stage, BUN, SAPS II, TET. For the selected variables, we performed univariate and multivariate Cox regression.

Restricted cubic spline was used to analyze the association between the HAR and endpoint events. We applied segmented regression (also known as piece-wise regression), which uses a separate line segment to fit each interval. Log-likelihood ratio test comparing one-line (non-segmented) model to segmented regression model was used to determine whether threshold exists. The inflection point which connects the segments was based on maximum likelihood that the model gives, and it was determined using the two-steps recursive method.

Interaction and stratified analyses were conducted based on age (≤65 and >65 years), sex, and comorbidity (asthma, diabetes, AP, AF).

All analyses were performed using the R software (version 4.2.2) and Empower Stats (http://www.empowerstats.com, X&Y Solutions, Inc.). A two-sided significance level of 0.05 was used to evaluate statistical significance.

### 2.5 Management of missing data

The number of missing data for WBC is 14, the number of missing data for platelets is 23, the number of missing data for Scr is 1, the number of missing data for INR is 162, the number of missing data for serum potassium is 8, the number of missing data for serum sodium is 1, the number of missing data for serum chloride is 2 and the number of missing data for serum total calcium is 16. The number of samples with missing data in this study is 162, only 3.1% of the total sample (S1 File). The number of samples with missing data is very small. So multiple imputation of missing data is not required.

### 2.6 Ethics approval and consent to participate

The data is publicly available (in the MIMIC-IV database). Therefore, the ethical approval statement and the requirement for Consent to Participate declaration were waived for this study.

## 3 Results

The median HAR of all patients included was 0.144 (IQR: 0.045–0.709). Patients were grouped into four groups based on the quartiles of the HAR [quartiles Q1 (0.045–0.12), Q2 (0.12–0.144), Q3 (0.144–0.179), Q4 (0.179–0.709)]. Mortality within 28 days after ICU admission was 34.29% (Table 1).

**Table 1:** Baseline characteristics of the four groups.

Categories	Q1(N = 1308)	Q2(N = 1264)	Q3(N = 1313)	Q4(N = 1295)	P-value
Age (years)	80.50 (72.46,87.24)	74.78 (66.91,82.36)	66.73 (59.75,74.05)	52.97 (42.04,60.52)	<0.001
Sex male	686 (52.45%)	654 (51.74%)	737 (56.13%)	792 (61.16%)	<0.001
Ethnicity					<0.001
Asian	44 (3.36%)	45 (3.56%)	45 (3.43%)	44 (3.40%)	
Black	142 (10.86%)	130 (10.28%)	145 (11.04%)	125 (9.65%)	
White	917 (70.11%)	918 (72.63%)	901 (68.62%)	789 (60.93%)	
Others	205 (15.67%)	171 (13.53%)	222 (16.91%)	337 (26.02%)	
Comorbidities					
Asthma	86 (6.57%)	93 (7.36%)	106 (8.07%)	142 (10.97%)	<0.001
Diabetes	490 (37.46%)	472 (37.34%)	516 (39.30%)	378 (29.19%)	<0.001
Acute pancreatitis	32 (2.45%)	30 (2.37%)	44 (3.35%)	78 (6.02%)	<0.001
Atrial fibrillation	612 (46.79%)	566 (44.78%)	463 (35.26%)	239 (18.46%)	<0.001
Acute myocardial infarction	7 (0.54%)	8 (0.63%)	16 (1.22%)	8 (0.62%)	0.158
AKI stage					0.002
Without AKI	258 (19.72%)	251 (19.86%)	267 (20.34%)	324 (25.02%)	
AKI stage 1	146 (11.16%)	131 (10.36%)	170 (12.95%)	163 (12.59%)	
AKI stage 2	406 (31.04%)	368 (29.11%)	374 (28.48%)	328 (25.33%)	
AKI stage 3	498 (38.07%)	514 (40.66%)	502 (38.23%)	480 (37.07%)	
Laboratory tests					
WBC, K/u L	12.90 (8.20,19.10)	13.50 (8.60,19.30)	14.20 (8.80,19.70)	13.80 (8.20,20.10)	0.137
Hemoglobin, g/dL	8.20 (7.30,9.00)	9.80 (8.70,10.80)	10.70 (9.50,11.80)	11.80 (10.20,13.10)	<0.001
Platelet, K/u L	186.00 (119.00,275.75)	188.00 (120.00,269.00)	184.00 (122.00,262.00)	175.50 (112.00,258.00)	0.060
Serum creatinine, mg/dL	1.50 (1.00,2.40)	1.40 (0.90,2.40)	1.40 (0.90,2.40)	1.30 (0.80,2.20)	<0.001
Blood Urea Nitrogen, mg/dL	35.00 (23.00,56.00)	31.00 (19.00,50.00)	28.00 (18.00,46.00)	24.00 (14.00,41.00)	<0.001
INR	1.40 (1.20,1.90)	1.40 (1.20,1.90)	1.40 (1.20,1.90)	1.40 (1.20,1.80)	0.014
Potassium, m Eq/L	4.10 (3.70,4.70)	4.10 (3.70,4.60)	4.10 (3.70,4.70)	4.10 (3.60,4.70)	0.382
Sodium, m Eq/L	138.00 (135.00,142.00)	138.00 (135.00,141.00)	137.00 (134.00,141.00)	137.00 (134.00,141.00)	<0.001
Chloride, m Eq/L	104.00 (100.00,109.00)	104.00 (99.00,109.00)	103.00 (98.00,108.00)	103.00 (98.00,108.00)	<0.001
Total Calcium, m Eq/L	7.90 (7.40,8.40)	8.00 (7.40,8.40)	8.00 (7.40,8.55)	7.80 (7.30,8.40)	<0.001
SOFA	3.00 (1.00,5.00)	3.00 (1.00,5.00)	3.00 (1.00,5.00)	3.00 (1.00,5.00)	0.528
SAPS II	67.00 (52.00,89.00)	67.00 (50.00,87.00)	67.00 (50.00,91.00)	66.00 (47.00,93.00)	0.554
Total erythrocyte transfusions within 27 days after ICU admission	342.69 (0.00–700.00)	0.00 (0.00–375.00)	0.00 (0.00–350.00)	0.00 (0.00–350.00)	<0.001
Mortality within 28 days after ICU admission	499 (38.15%)	427 (33.78%)	423 (32.22%)	427 (32.97%)	0.006
HAR	0.11 (0.10,0.11)	0.13 (0.13,0.14)	0.16 (0.15,0.17)	0.22 (0.19,0.26)	<0.001

### 3.1 Baseline characteristics

Compared with the other three groups, patients in the lowest quartile of the HAR had a higher prevalence of age, TET, Scr, BUN and AF and a lower prevalence of Hb and asthma. Patients with lowest quartiles of the HAR had highest mortality (Table 1).

Survivors had higher HAR, Hb and length of stays (LOS) in the hospital than non-survivors. Survivors had a lower prevalence of BUN, SOFA, TET, LOS in the ICU than non-survivors (Table 2).

**Table 2:** Baseline characteristics of survivors and non-survivors.

Categories	Survivor(N = 3404)	Non-survivor(N = 1776)	P-value
Age (years)	69.35 (58.68,79.78)	68.45 (57.94,79.80)	0.138
Sex male	1869 (54.91%)	1000 (56.31%)	0.336
Ethnicity			0.719
Asian	111 (3.26%)	67 (3.77%)	
Black	355 (10.43%)	187 (10.53%)	
White	2314 (67.98%)	1211 (68.19%)	
Others	624 (18.33%)	311 (17.51%)	
Comorbidities			
Asthma	274 (8.05%)	153 (8.61%)	0.482
Diabetes	1224 (35.96%)	632 (35.59%)	0.791
Acute pancreatitis	115 (3.38%)	69 (3.89%)	0.350
Atrial fibrillation	1254 (36.84%)	626 (35.25%)	0.258
Acute myocardial infarction	31 (0.91%)	8 (0.45%)	0.069
AKI stage			0.032
Without AKI	762 (22.39%)	338 (19.03%)	
AKI stage 1	404 (11.87%)	206 (11.60%)	
AKI stage 2	945 (27.76%)	531 (29.90%)	
AKI stage 3	1293 (37.98%)	701 (39.47%)	
Laboratory tests			
WBC, K/u L	13.60 (8.40,19.60)	13.50 (8.40,19.60)	0.866
Hemoglobin, g/dL	10.10 (8.70,11.60)	9.70 (8.20,11.20)	<0.001
Platelet, K/u L	184.00 (119.00,264.00)	183.00 (116.00,268.00)	0.804
Serum creatinine, mg/dL	1.40 (0.90,2.30)	1.40 (0.90,2.40)	0.663
Blood Urea Nitrogen, mg/dL	29.00 (18.00,48.00)	30.00 (19.00,50.00)	0.007
INR	1.40 (1.20,1.90)	1.40 (1.20,1.90)	0.635
Potassium, m Eq/L	4.10 (3.60,4.70)	4.10 (3.60,4.68)	0.382
Sodium, m Eq/L	138.00 (134.00,141.00)	138.00 (134.00,141.00)	0.231
Chloride, m Eq/L	104.00 (99.00,108.00)	104.00 (99.00,109.00)	0.285
Total Calcium, m Eq/L	7.90 (7.40,8.50)	7.90 (7.40,8.50)	0.954
SAPS.II	3.00 (1.00,5.00)	3.00 (1.00,5.00)	0.506
SOFA	65.00 (49.00,89.00)	69.00 (51.00,91.00)	0.011
TET	0.00 (0.00,350.00)	350.00 (0.00,713.75)	<0.001
LOS in hospital, days	12.51 (7.10,22.86)	8.37 (3.84,14.61)	<0.001
LOS in ICU, days	3.70 (2.10,8.09)	4.42 (2.24,8.64)	0.008
HAR	0.15 (0.12,0.18)	0.14 (0.12,0.18)	<0.001

### 3.2 Primary results

The results of Cox regression and Cox proportional hazards modeling showed that the HAR was a protective factor for death within 28 days after ICU admission in both unadjusted (model 1), partially adjusted (model 2) and completely adjusted models (model 3), HRs were 0.31 (95% CI: 0.14, 0.68), 0.31 (95% CI: 0.14, 0.69) and 1.125 (95% CI: 0.14, 0.72), respectively (S2 File, Table 3).

**Table 3:** Cox proportional hazards modeling.

Categories	Model 1		Model 2		Model 3	
	HR (95% CI)	P-value	HR (95% CI)	P-value	HR (95% CI)	P-value
All-cause mortality						
Continuous variable per unit	0.31 (0.14, 0.68)	0.0037	0.31 (0.14, 0.69)	0.004	0.31 (0.14, 0.72)	0.0065
Quartiles						
Q1 (N = 1308)	Ref		Ref		Ref	
Q2 (N = 1264)	0.86 (0.76, 0.98)	0.0273	0.87 (0.76, 0.98)	0.0281	0.88 (0.77, 1.00)	0.0548
Q3 (N = 1313)	0.82 (0.72, 0.93)	0.0021	0.82 (0.72, 0.93)	0.0021	0.84 (0.74, 0.96)	0.0108
Q4 (N = 1295)	0.85 (0.75, 0.97)	0.0161	0.85 (0.75, 0.97)	0.0179	0.86 (0.75, 0.99)	0.0357

Model 1: Unadjusted.

Model 2: Adjusted for sex.

Model 3: Adjusted for sex, AF, AMI, AKI stage, BUN, SAPS II, TET.

**Fig 3:**
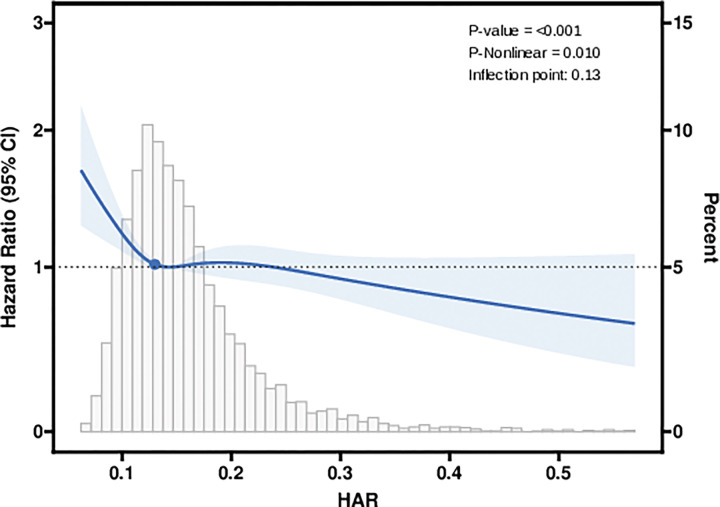
Restricted cubic spline for mortality within 28 days after ICU admission adjusted for sex, AF, AMI, AKI stage, BUN, SAPS II, TET. Heavy central lines represent the estimated adjusted hazard ratios, with shaded ribbons denoting 95% confidence intervals.

Restricted cubic spline showed a nonlinear association between the HAR and mortality within 28 days after ICU admission, after adjustment for covariates (Fig 3).

Threshold effect analysis showed that, after adjustment for covariates, when the HAR was lower than 0.13, mortality within 28 days after ICU admission decreased significantly as the HAR increased, and the HR was 0.0 (95% CI: 0.0, 0.1). However, when the HAR was higher than 0.13, the HAR might not be a protective factor for mortality within 28 days after ICU admission (P = 0.235) (Table 4).

**Table 4:** Threshold effect analysis of the HAR on mortality within 28 days after ICU admission.

		Adjusted HR (95%CI)	P-value
Fitting by standard linear model	HAR	0.3 (0.1, 0.6)	0.003
Fitting by two-piecewise linear model			
	Inflection point	0.13	
	HAR < 0.13	0.0 (0.0, 0.1)	<0.001
	HAR > 0.13	0.6 (0.2, 1.4)	0.235
	Log likelihood ratio		0.006

**Fig 4:**
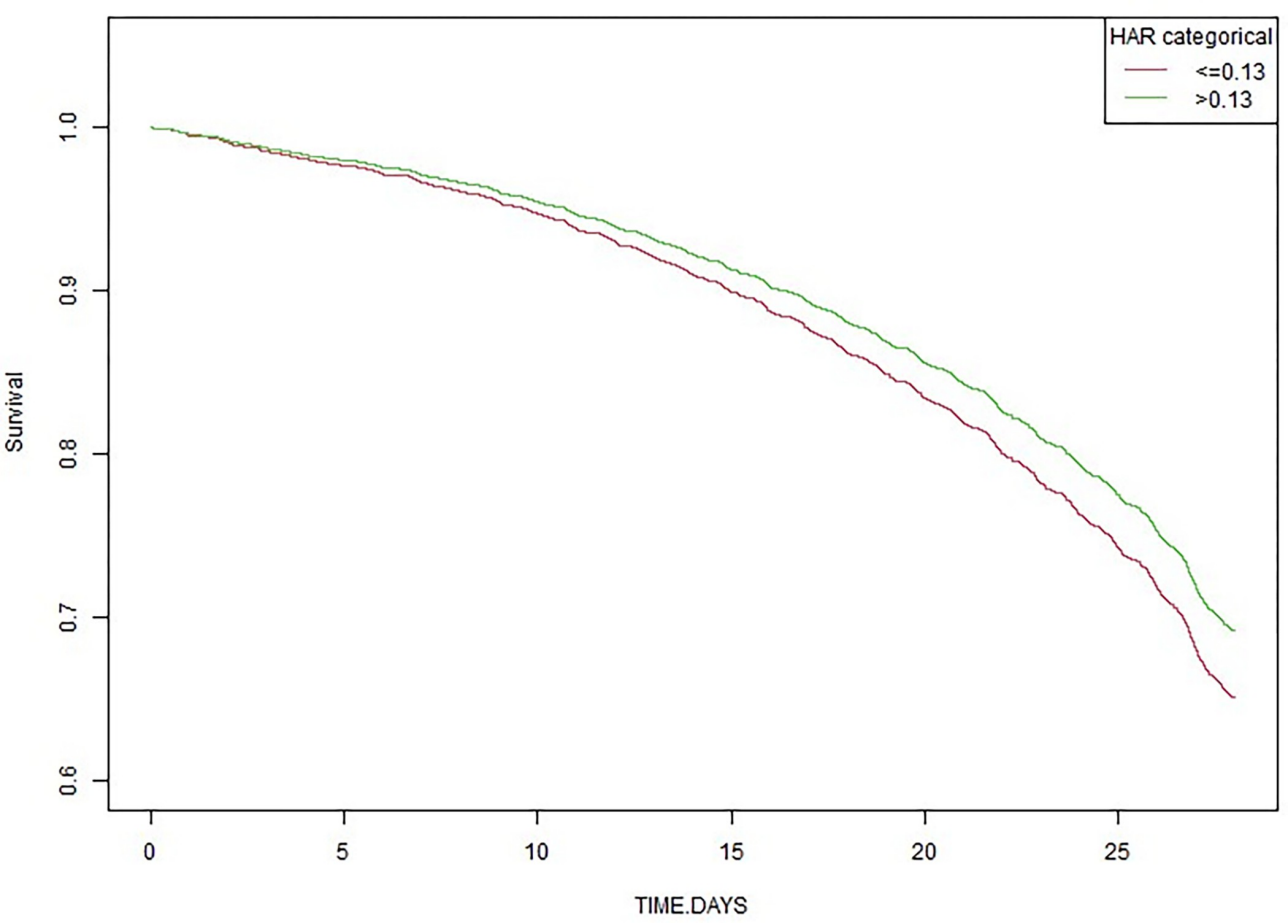
Kaplan-Meier curves showing cumulative survival of different groups of patients within 28 days after ICU admission.

The results of the Kaplan-Meier survivor analysis showed that, after adjustment for sex, AF, AMI, AKI stage, BUN, SAPS II, TET, patients with the HAR above 0.13 had a higher cumulative survival within 28 days after ICU admission, compared to patients with the HAR below 0.13 (Fig 4).

Spearman’s rank correlation analysis showed a negative correlation between HAR and mortality within 28 days after ICU admission in the overall patients with SS (P<0.001). The results of segmented correlation analysis showed that, when the HAR was lower than 0.13, a negative correlation between HAR and mortality within 28 days after ICU admission (P = 0.020). When the HAR was lower than 0.13, there was no correlation between HAR and mortality within 28 days after ICU admission (P = 0.9776) (Table 5).

**Table 5:** Correlation coefficients between Hemoglobin-to-Age Ratio and mortality in patients with SS within 28 days after ICU admission.

Categories	Correlation coefficients	P-value	Numbers of patients
All patients	-0.0472	<0.001	5180
HAR < = 0.13	-0.0536	0.0201	1881
HAR > 0.13	0.0005	0.9776	3299

### 3.3 Subgroup analysis

**Fig 5:**
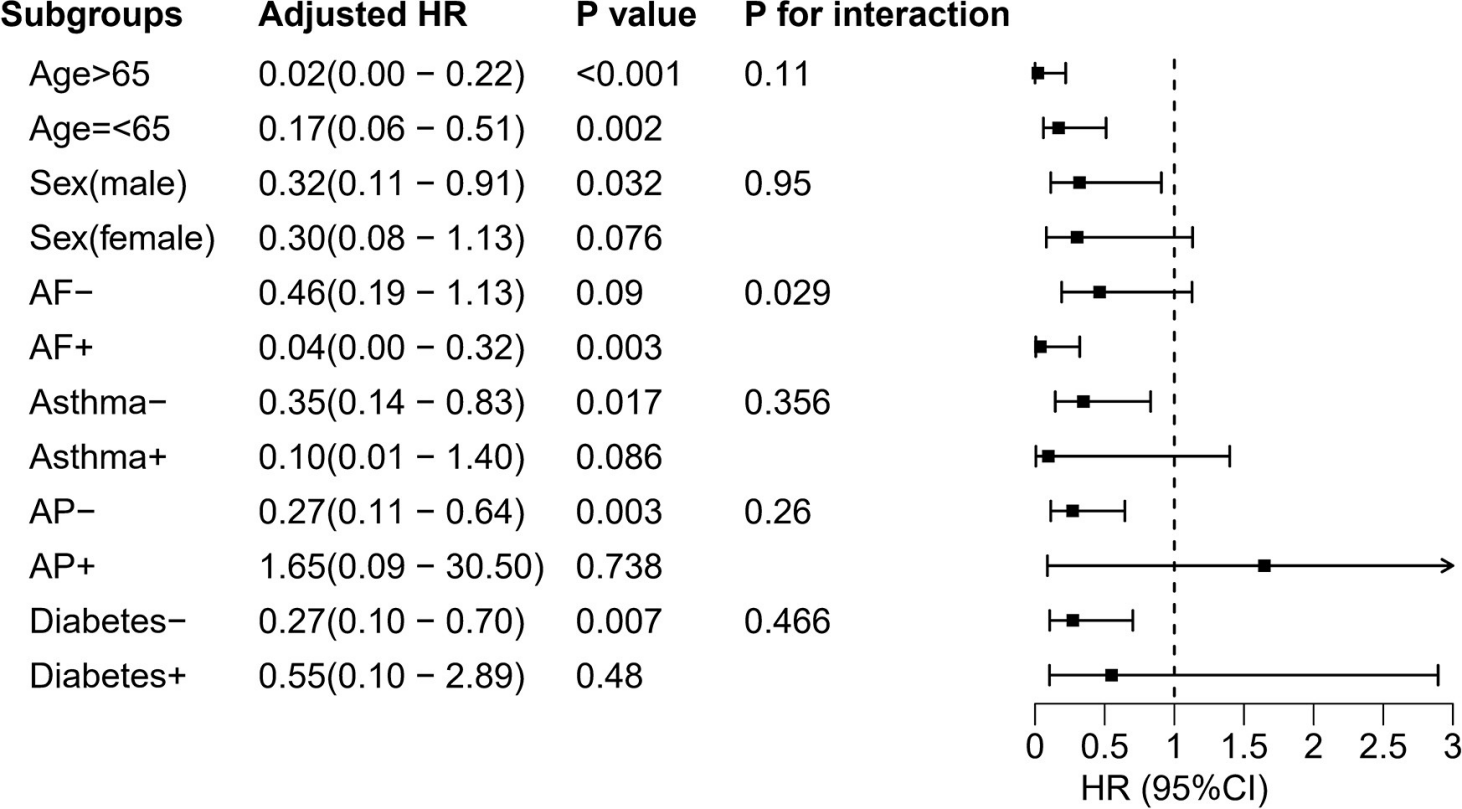
Forest plots of hazard ratios for the mortality within 28 days after ICU admission in different subgroups, adjusted for sex, AF, AMI, AKI stage, BUN, SAPS II, TET.

The interaction analysis revealed that there was a significant interaction between AF and HAR in predicting the risk of death within 28 days after ICU admission. After adjustment for covariates, the effect of the HAR on reducing death within 28 days after ICU admission remained more significant in patients with AF than in patients without AF [HR = 0.038 (95% CI: 0.005, 0.321) vs. HR = 0.464 (95% CI: 0.191, 1.127), P for interaction = 0.029] (Fig 5).

## 4 Discussion

In our study, we excluded SS patients with bleeding comorbidities and adjusted for the confounding variable of total erythrocyte transfusion (TET) within 28 days after ICU admission. The results of the study showed that the HAR might be a protective factor in reducing all-cause mortality in patients with SS within 28 days after ICU admission. Threshold effect analyses showed that when the HAR was lower than 0.13, mortality within 28 days after ICU admission decreased significantly with increasing HAR; however, when HAR was greater than 0.13, changes in HAR had no effect on mortality within 28 days after ICU admission. This phenomenon suggests that patients with SS of different ages have different hemoglobin requirements. In patients with SS whose HAR was lower than 0.13, the hemoglobin requirement was higher in older patients. However, in patients with SS whose HAR was higher than 0.13, the hemoglobin requirement may no longer be affected by age.

Hb concentration is closely associated with the prognosis of sepsis. Oxygen delivery depends mainly on Hb concentration, cardiac output, and oxygen saturation [9,10]. A decrease in Hb concentration leads to a reduction in oxygen delivery [11]. When the body’s demand for oxygen is greater than oxygen delivery, tissue cells undergo anaerobic glycolysis and produce lactic acid [12,13]. Persistent hyperlactatemia is strongly associated with an elevated risk of death in patients with sepsis [14]. Other studies have shown that anemia may impair cardiac function and induce heart failure [15,16]. Studies have shown that anemia increases mortality in patients with sepsis [11,17]. Previous studies have analyzed the effect of transfusion threshold on mortality in patients with SS. The results showed no significant difference in mortality between patients with SS whose transfusion threshold was 7 g/dL and those whose transfusion threshold was 9 g/dL. Blood transfusion may improve the prognosis of patients with SS only when the hemoglobin concentration is below 7 g/dL [1,12,18,19]. Recent studies have shown that hemoglobin concentration varies with age [7]. Therefore, basic hemoglobin and oxygen delivery levels may vary in patients with SS of different ages. Yang P et al. showed a nonlinear relationship between hemoglobin concentration and the 28-day mortality risk in elderly patients with sepsis. Both hemoglobin concentrations below 10 g/dL and above 15 g/dL increased the 28-day mortality risk in patients with sepsis [20]. Zhu YB et al. found that low levels of hemoglobin concentration were an independent risk factor for poor prognosis of sepsis in pediatric patients with sepsis [21]. The above findings may suggest that the requirement for hemoglobin concentration may not be the same in patients with sepsis of different ages.

Several studies have demonstrated that advanced age is an essential factor contributing to the elevated risk of death in sepsis and SS [5,22–24]. Loue S et al. observed T-cell depletion in septic-aged mice (aged between 20 and 22 months). Also, interleukin 6 was at persistently high levels in both elderly patients (age >65 years) and elderly mice (age between 20 months and 22 months) with sepsis [25]. Saito H et al. also observed significantly elevated levels of interleukin 6 in elderly mice with sepsis (age was 24 months) [26]. Michels EHA et al. showed that, compared to the younger patients with sepsis, there was significant dysfunction of endothelial cells in older patients with sepsis [27]. Epidemiological findings have shown that the prevalence of anemia is progressively increasing in the older people [28]. In elderly patients with anemia, especially those with HAR below 0.13, aggressive improvement of hemoglobin concentration may be able to compensate for the detrimental effects of advanced age on patients with SS. Although transfusion of erythrocyte can increase the hemoglobin concentration of patients. However, transfusion of large amounts of erythrocyte may induce transfusion-related acute lung injury [29–34], febrile nonhemolytic transfusion reaction, acute hemolytic transfusion reaction, allergic and anaphylactic transfusion reactions, transfusion-associated circulatory overload, septic transfusion reaction, hypotensive transfusion reaction, delayed hemolytic transfusion reactions, transfusion-associated graft-versus-host disease and so on [35–37]. Therefore, when the HAR of a patient with SS is higher than 0.13, the patient may not benefit from transfusion of erythrocyte.

Stratified analyses showed that the protective effect of HAR was more pronounced in patients with AF compared with those without AF. Previous studies have shown that AF is strongly associated with the development of heart failure [38–40]. Patients with AF have poorer basic cardiac function compared with those without AF. As mentioned earlier, oxygen delivery is affected by both Hb concentration and cardiac output. Therefore, among patients with SS of the same age group, patients with AF may have a higher Hb requirement than those without AF.

This study also has some limitations. Restricted cubic spline showed that when the HAR was below 0.13, mortality within 28 days after ICU admission decreased significantly with increasing HAR. Spearman’s rank correlation analysis showed that when HAR was below 0.13, there was a negative correlation between HAR and mortality within 28 days after ICU admission. However, the correlation was weak. The reason for these results may be that the prognosis of SS is influenced by a combination of many factors. The correlation analysis did not exclude the interference of other confounding variables on the prognosis of SS. Therefore, the relationship between HAR and the prognosis of SS still needs to be further validated in prospective randomized controlled studies.

## 5 Conclusion

In conclusion, there was a nonlinear association between the HAR and mortality within 28 days after ICU admission. When the HAR was at a low level, mortality within 28 days after ICU admission decreased significantly as the HAR increased. When the HAR was at high levels, the HAR might not be a protective factor for mortality within 28 days after ICU admission. In patients with SS, the HAR was more effective in reducing the risk of death in patients with AF than in patients without AF.

## Supporting information

S1 Raw data Raw data of this study. (XLS)

S1 File Percentage of missing values. (DOCX)

S2 File Cox regression. (DOCX)

S1 Fig Scatterplot 1. (JPG)

S2 Fig Scatterplot 2. (JPG)

S3 Fig Scatterplot 3. (JPG)

S4 Fig Scatterplot 4. (JPG)

S5 Fig Scatterplot 5. (JPG)

S6 Fig Scatterplot 6. (JPG)

S7 Fig Scatterplot 7. (JPG)

S8 Fig Scatterplot 8. (JPG)

S9 Fig Scatterplot 9. (JPG)

## Abbreviations

SOFA: Sequential Organ Failure Assessment
SIRS: Systemic Inflammatory Response Syndrome
NEWS: National Early warning Score
MEWS: Modified Early warning Score
HAR: Age-to-Hb Ratio
MIMIC-IV: Medical Information Mart for Intensive Care IV
ICU: Intensive care unit
ICD: International Classification of Diseases
SQL: Structured Query Language
AP: Acute pancreatitis
AF: Atrial fibrillation
AMI: Acute myocardial infarction
AKI: Acute kidney injury
WBC: White blood cell
Hb: Hemoglobin
Scr: Serum creatinine
BUN: Blood Urea Nitrogen
PTT: Partial thromboplastin time
INR: International Normalized Ratio
SAPS II: Simplified Acute Physiology Score II
TET: Total erythrocyte transfusion
HR: Hazard ratios
CI: confidence intervals
IQR: Interquartile range
LOS: Length of stays
