# Supplementary material for: Nonlinear relationship between Hemoglobin-to-Age Ratio and all-cause mortality in patients with septic shock: A retrospective cohort study in the MIMIC-IV database
Source: PLoS One. 2024 Dec 6;19(12):e0313937. doi: 10.1371/journal.pone.0313937 (PMC11623482; doi:10.1371/journal.pone.0313937)
Supplement: S1 File — (DOCX) [file pone.0313937.s002.docx]

| **Percentage of missing values for variables** | |  |
| --- | --- | --- |
| Variables | Missing values (%) |  |
| Age（years） | 0 |  |
| Sex male | 0 |  |
| Ethnicity |  |  |
| Asian | 0 |  |
| Black | 0 |  |
| White | 0 |  |
| Others | 0 |  |
| Commorbidities |  |  |
| Asthma | 0 |  |
| Diabetes | 0 |  |
| Acute pancreatitis | 0 |  |
| Atrial fibrillation | 0 |  |
| Acute myocardial infarction | 0 |  |
| AKI stage | 0 |  |
| Laboratory tests |  |  |
| WBC,K/uL | 14(0.27%) |  |
| Hemoglobin,g/dL | 0 |  |
| Platelet,K/uL | 23(0.44%) |  |
| Serum creatinine,mg/dL | 1(0.02%) |  |
| Blood Urea Nitrogen,mg/dL | 0 |  |
| INR | 162(3.13%) |  |
| Potassium,mEq/L | 8(0.15%) |  |
| Sodium,mEq/L | 1(0.02%) |  |
| Chloride,mEq/L | | 2(0.04%) |
| Total Calcium,mEq/L | 16(0.31%) |  |
| SAPS.II | 0 |  |
| SOFA | 0 |  |
| HAR | 0 |  |
