# Supplementary material for: Nonlinear relationship between Hemoglobin-to-Age Ratio and all-cause mortality in patients with septic shock: A retrospective cohort study in the MIMIC-IV database
Source: PLoS One. 2024 Dec 6;19(12):e0313937. doi: 10.1371/journal.pone.0313937 (PMC11623482; doi:10.1371/journal.pone.0313937)
Supplement: S2 File — (DOCX) [file pone.0313937.s003.docx]

| Univariate and multivariate analysis of influencing factors (Cox regression) | | | | | | | | | | | | | |
| --- | --- | --- | --- | --- | --- | --- | --- | --- | --- | --- | --- | --- | --- |
| **Characteristic** | **Univariable** | | | | | | **Multivariable** | | | | | | |
|  | **N** | **Event N** | **Estimate** | **SE1** | **95% CI1** | **p-value** | **N** | **Event N** | **Estimate** | **SE1** | **95% CI1** | **p-value** |  |
| **HAR** | 5,180 | 1,776 | 1.1693450352 | 0.4034344252 | 0.1408496910, 0.6848002482 | 0.004 | 5,180 | 1,776 | 1.2598438603 | 0.4190874100 | 0.1247753835, 0.6450369801 | 0.003 |  |
| **Sex** |  |  |  |  |  |  |  |  |  |  |  |  |  |
| 1 | 2,869 | 1,000 |  |  |  |  | 2,869 | 1,000 |  |  |  |  |  |
| 2 | 2,311 | 776 | 0.0397180960 | 0.0478401271 | 0.8750423252, 1.0555340318 | 0.406 | 2,311 | 776 | 0.0350359635 | 0.0485319262 | 0.8779577644, 1.0619266620 | 0.470 |  |
| **AF** |  |  |  |  |  |  |  |  |  |  |  |  |  |
| 0 | 3,300 | 1,150 |  |  |  |  | 3,300 | 1,150 |  |  |  |  |  |
| 1 | 1,880 | 626 | 0.0505839460 | 0.0496693834 | 0.8624879090, 1.0478770461 | 0.308 | 1,880 | 626 | 0.1175907164 | 0.0516961862 | 0.8033913053, 0.9838635569 | 0.023 |  |
| **AMI** |  |  |  |  |  |  |  |  |  |  |  |  |  |
| 0 | 5,141 | 1,768 |  |  |  |  | 5,141 | 1,768 |  |  |  |  |  |
| 1 | 39 | 8 | 0.6168329486 | 0.3543538017 | 0.2694550649, 1.0807851207 | 0.082 | 39 | 8 | 0.6340306029 | 0.3545388727 | 0.2647646331, 1.0627424870 | 0.074 |  |
| **AKI stage** |  |  |  |  |  |  |  |  |  |  |  |  |  |
| 0 | 1,100 | 338 |  |  |  |  | 1,100 | 338 |  |  |  |  |  |
| 1 | 610 | 206 | 0.1068942742 | 0.0883911880 | 0.9358039581, 1.3233121777 | 0.227 | 610 | 206 | 0.0850881636 | 0.0894758318 | 0.9136741835, 1.2975236608 | 0.342 |  |
| 2 | 1,476 | 531 | 0.1988320839 | 0.0695852137 | 1.0644386502, 1.3982432072 | 0.004 | 1,476 | 531 | 0.1739303319 | 0.0712103216 | 1.0349578138, 1.3682054593 | 0.015 |  |
| 3 | 1,994 | 701 | 0.1663468900 | 0.0662212903 | 1.0372319165, 1.3446560211 | 0.012 | 1,994 | 701 | 0.1083717524 | 0.0747200362 | 0.9626389809, 1.2902297875 | 0.147 |  |
| **BUN** | 5,180 | 1,776 | 0.0020856362 | 0.0008210333 | 1.0004765540, 1.0037016662 | 0.011 | 5,180 | 1,776 | 0.0016197778 | 0.0008993500 | 0.9998570944, 1.0033881985 | 0.072 |  |
| **SAPSII** | 5,180 | 1,776 | 0.0016904081 | 0.0008121984 | 1.0000985335, 1.0032876803 | 0.037 | 5,180 | 1,776 | 0.0004745913 | 0.0009806837 | 0.9985535337, 1.0023995704 | 0.628 |  |
| **TET** | 5,180 | 1,776 | 0.0000441767 | 0.0000100352 | 1.0000245082, 1.0000638474 | <0.001 | 5,180 | 1,776 | 0.0000420678 | 0.0000105779 | 1.0000213358, 1.0000628021 | <0.001 |  |
| 1SE = Standard Error, CI = Confidence Interval | | | | | | | | | | | | | |

The AIC values for each model after we tried modelling with different knot numbers (3, 4, 5, 6, 7) are 29660.0727196325, 29656.8775100301, 29658.6321443836, 29660.5885573276, 29663.0665820505

Number of Knot nodes automatically selected based on minimum AIC value: 4

These Knot nodes are located at the 5th, 35th, 65th and 95th percentile of the values of all patient independent variables, respectively.
