# Supplementary figures and images for: Nonlinear relationship between Hemoglobin-to-Age Ratio and all-cause mortality in patients with septic shock: A retrospective cohort study in the MIMIC-IV database
Source: PLoS One. 2024 Dec 6;19(12):e0313937. doi: 10.1371/journal.pone.0313937 (PMC11623482; doi:10.1371/journal.pone.0313937)

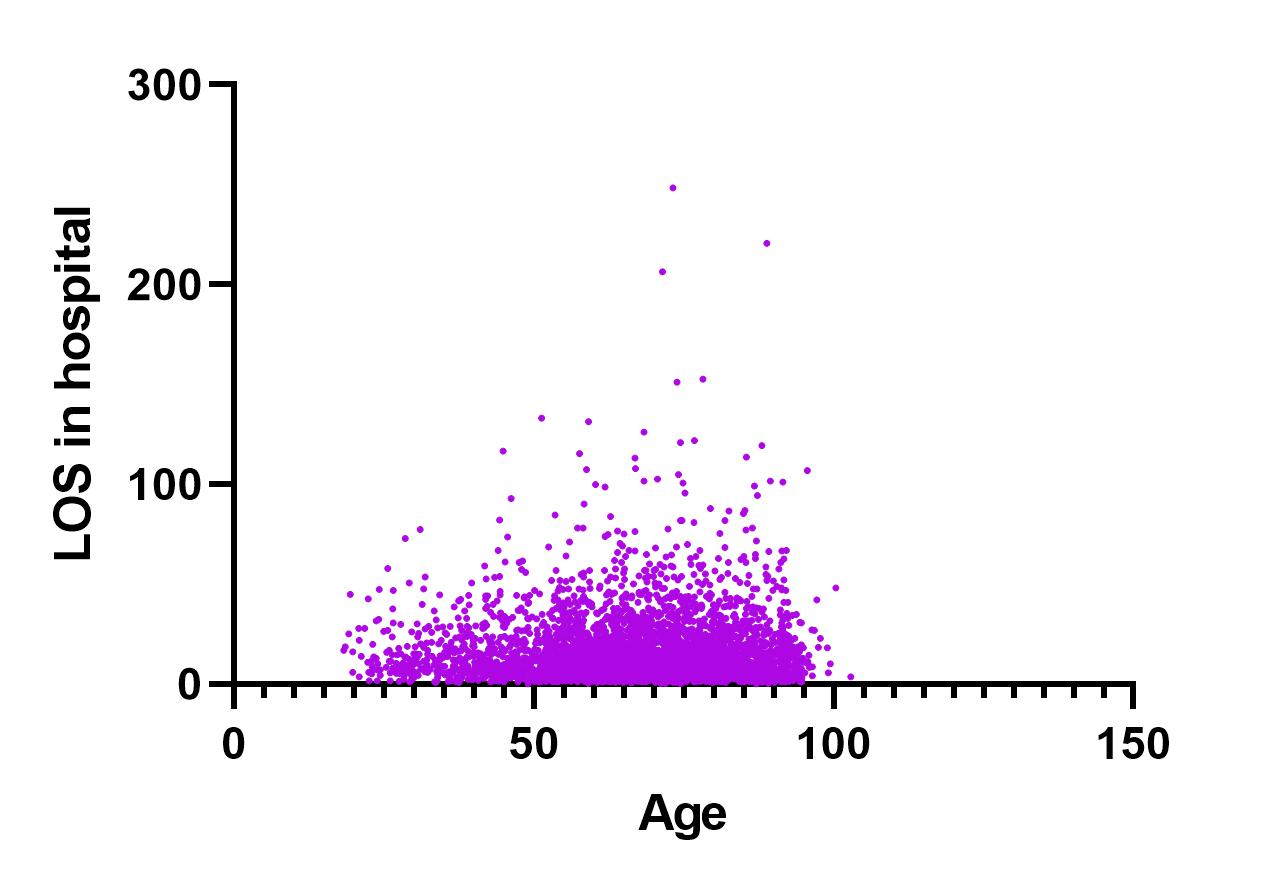

Supplement: S1 Fig — (JPG) [file pone.0313937.s004.jpg]

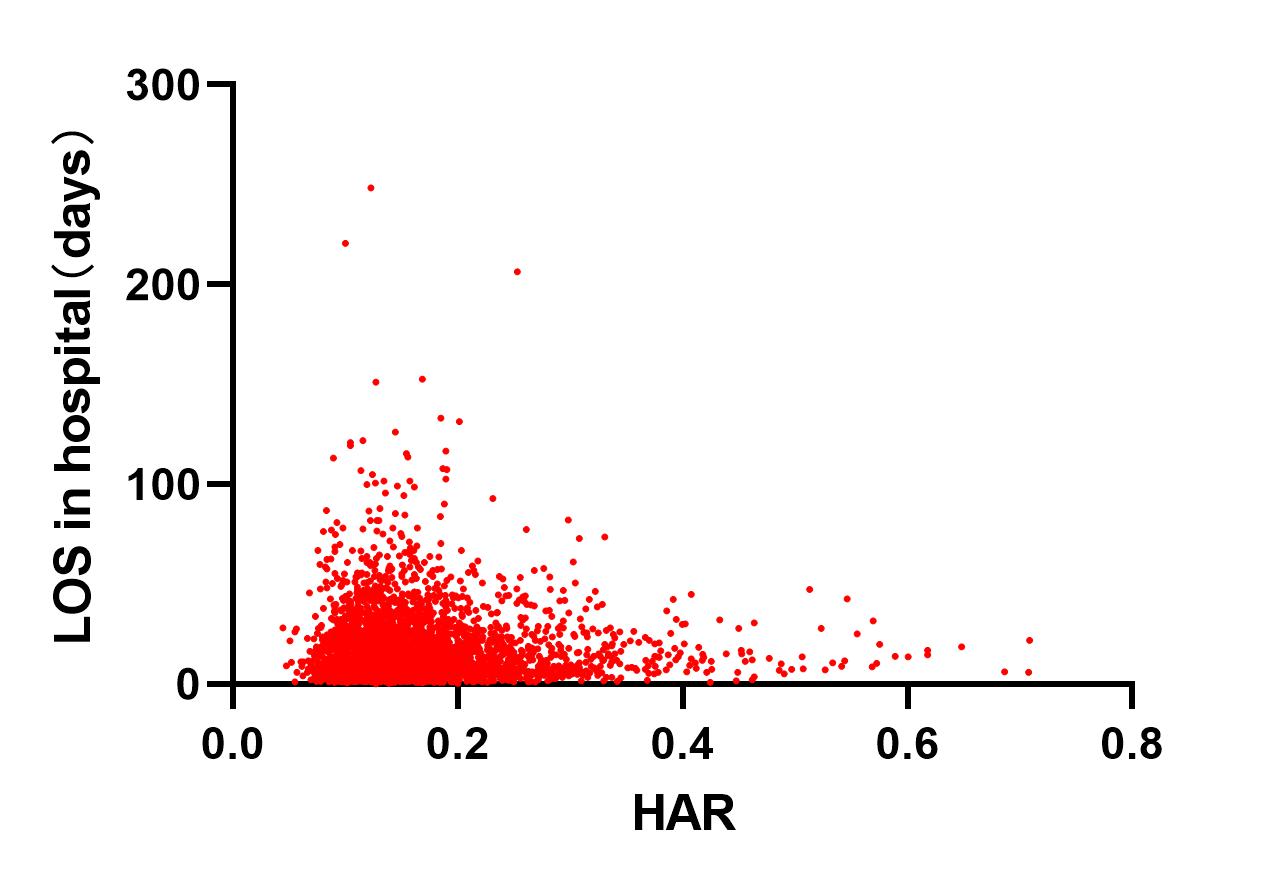

Supplement: S2 Fig — (JPG) [file pone.0313937.s005.jpg]

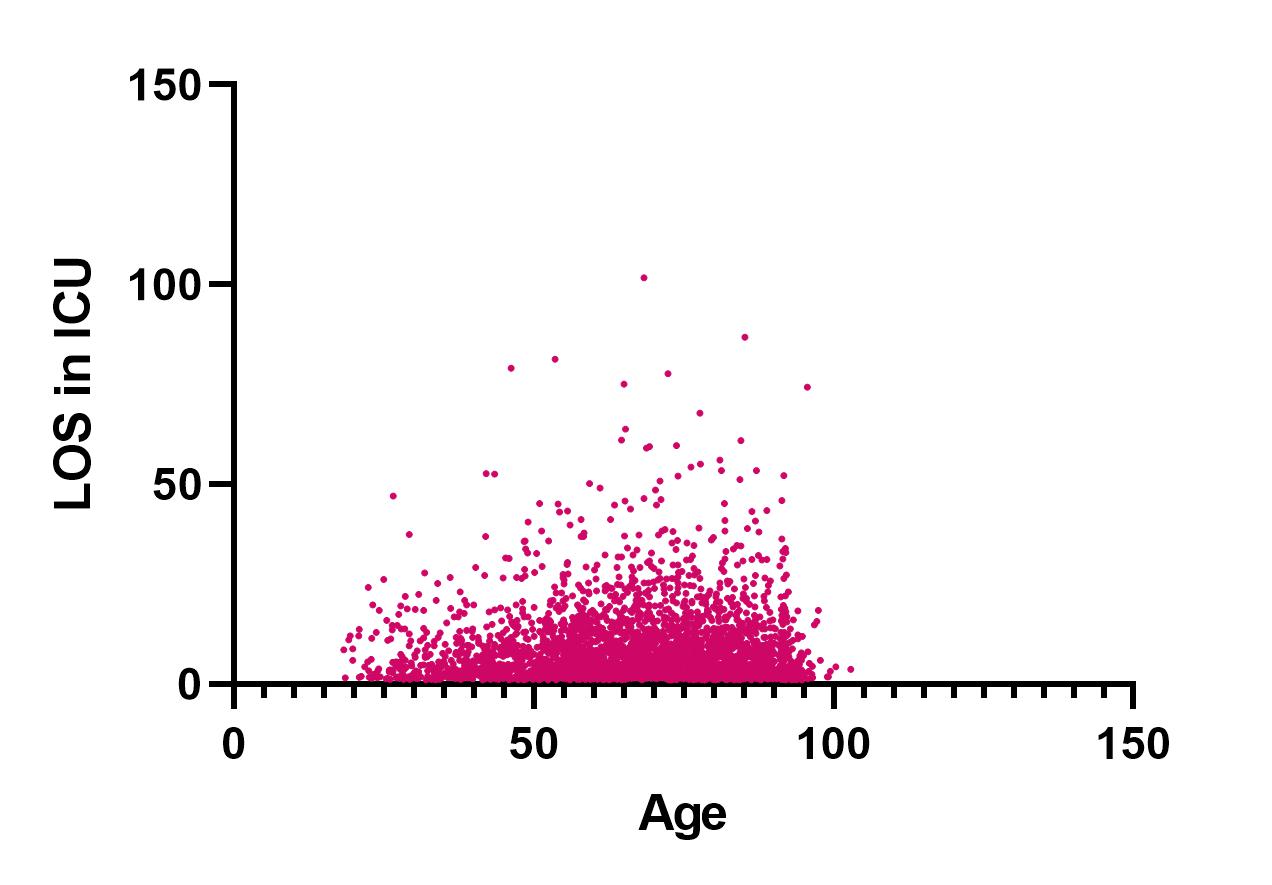

Supplement: S3 Fig — (JPG) [file pone.0313937.s006.jpg]

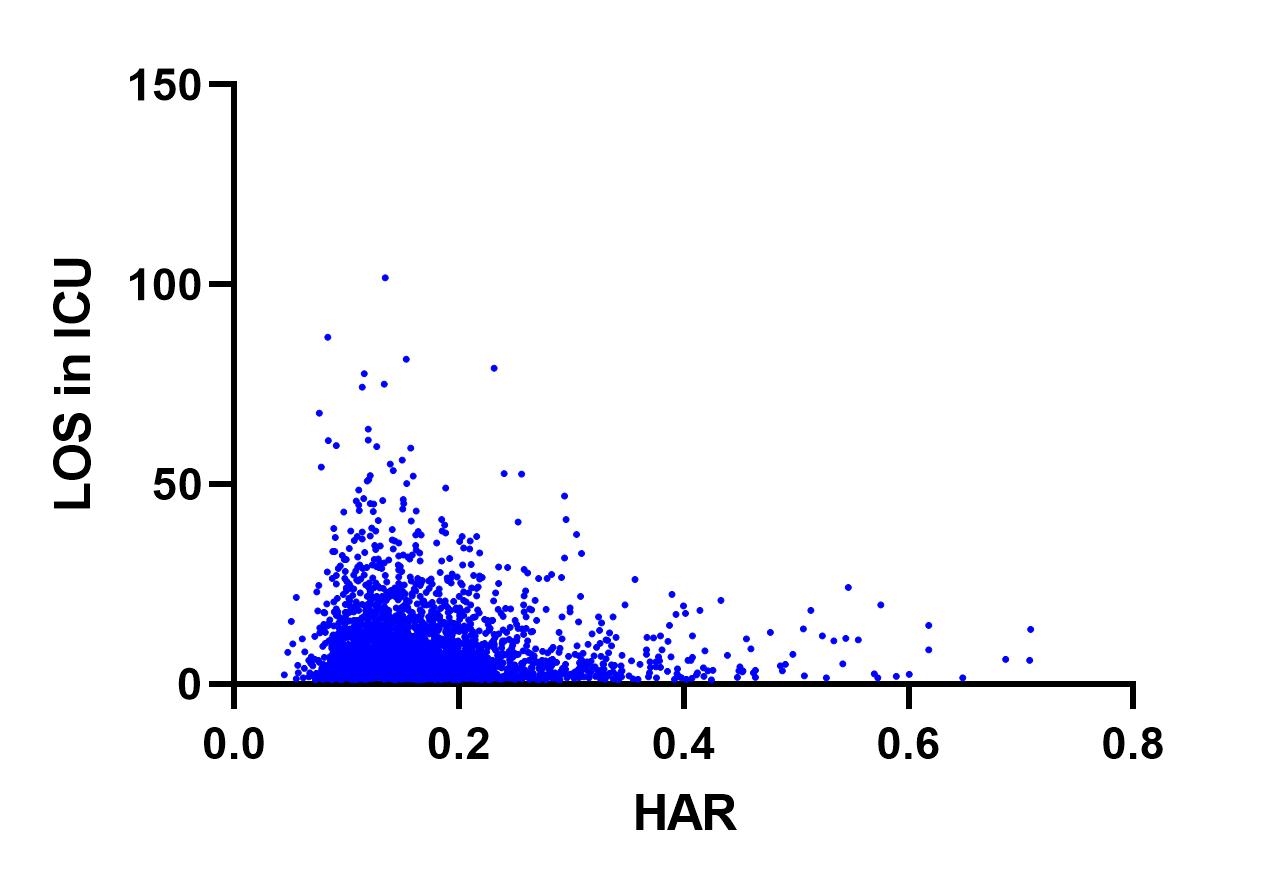

Supplement: S4 Fig — (JPG) [file pone.0313937.s007.jpg]

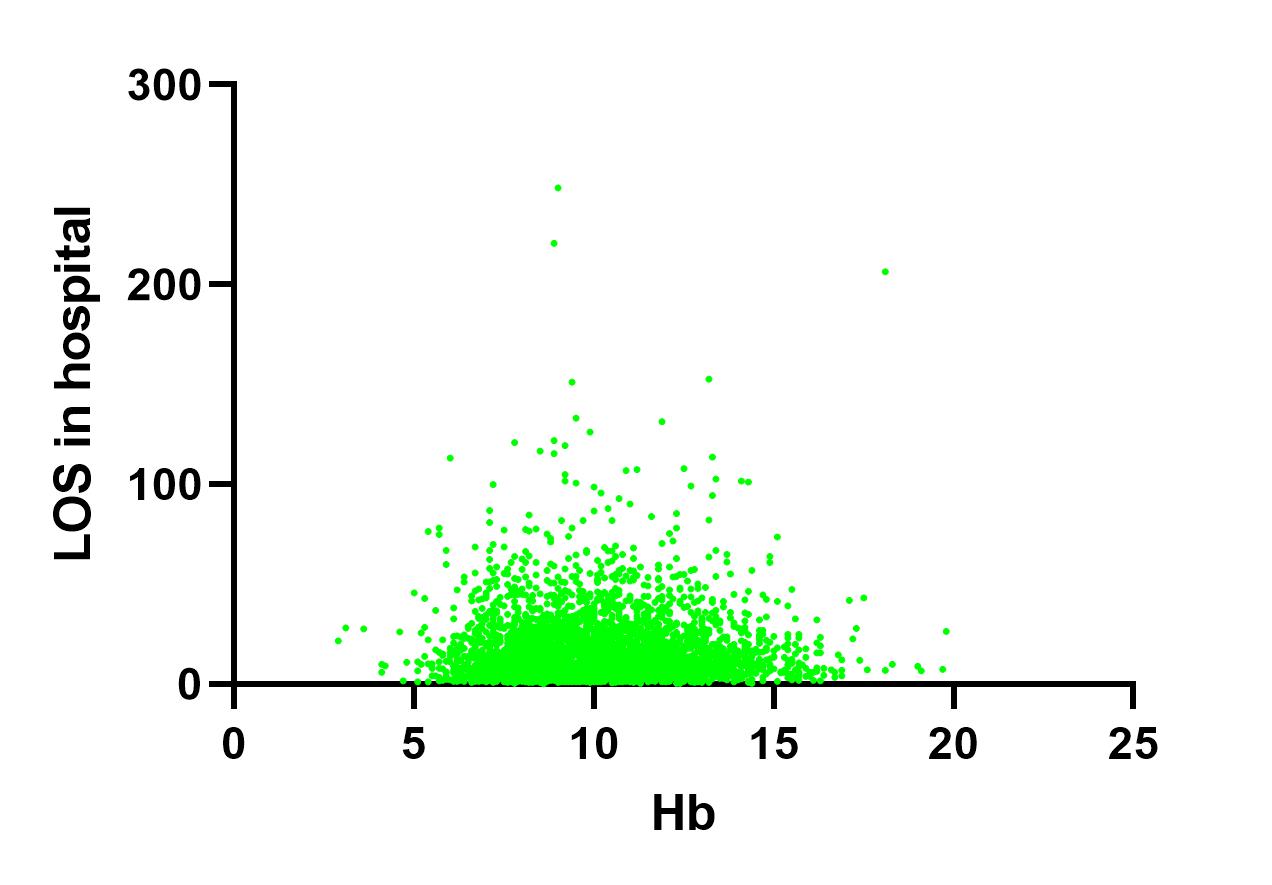

Supplement: S5 Fig — (JPG) [file pone.0313937.s008.jpg]

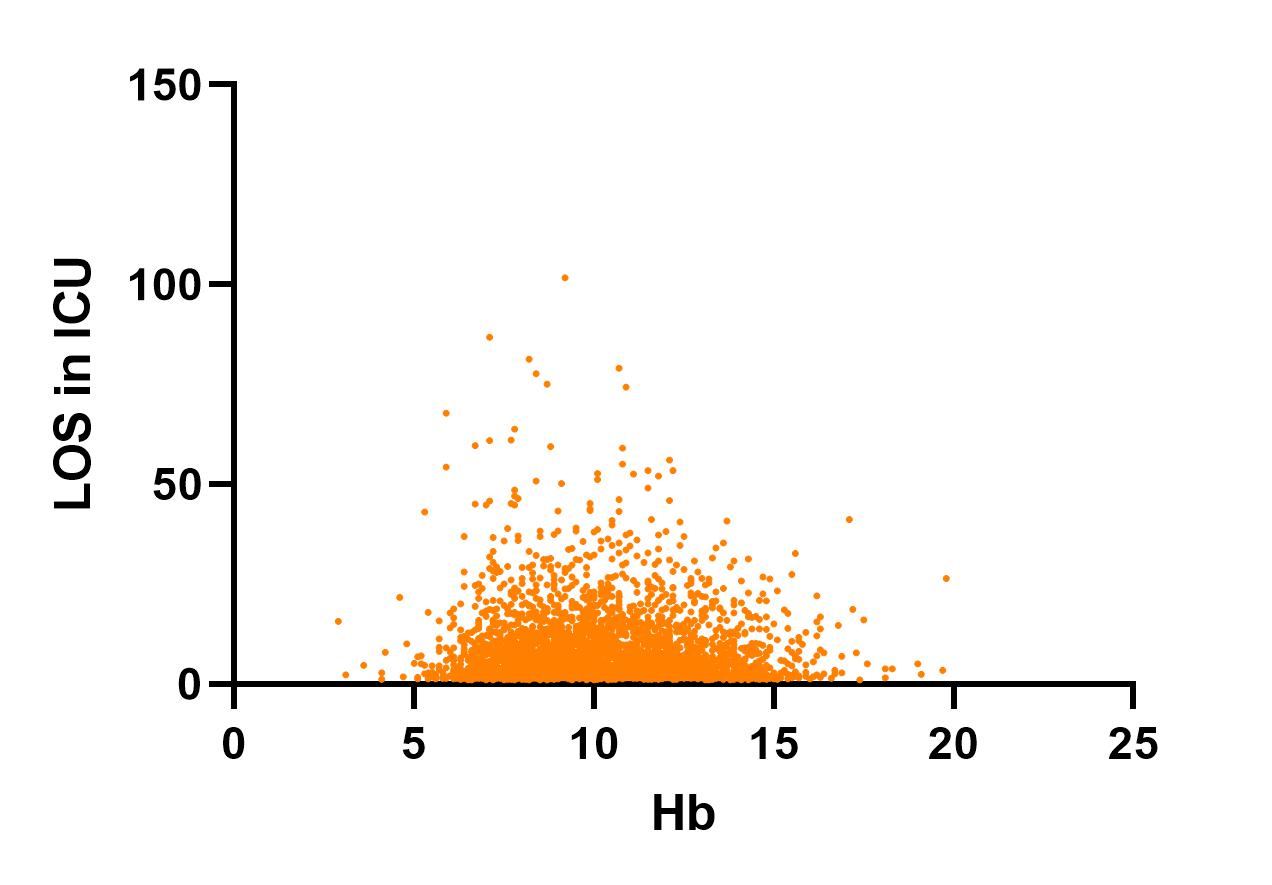

Supplement: S6 Fig — (JPG) [file pone.0313937.s009.jpg]

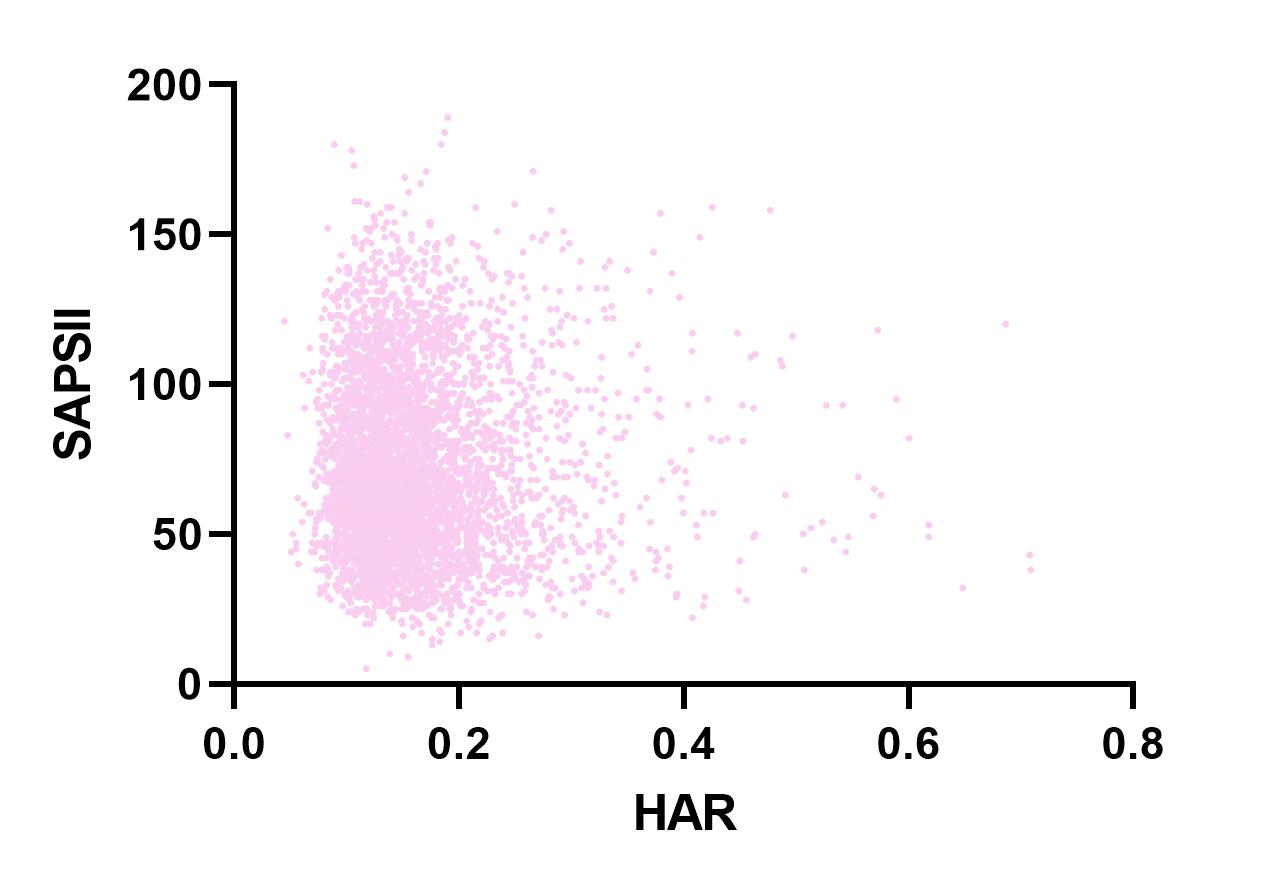

Supplement: S7 Fig — (JPG) [file pone.0313937.s010.jpg]

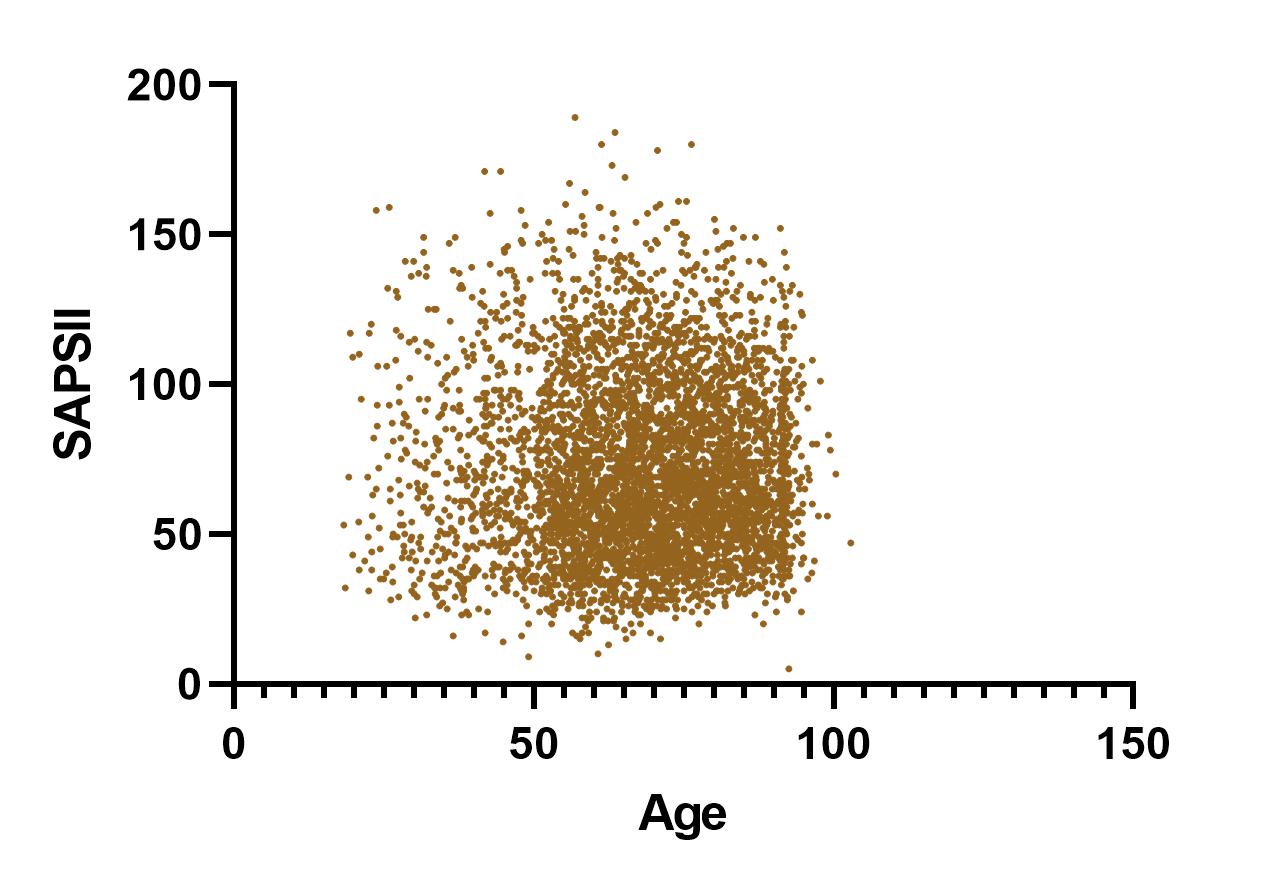

Supplement: S8 Fig — (JPG) [file pone.0313937.s011.jpg]

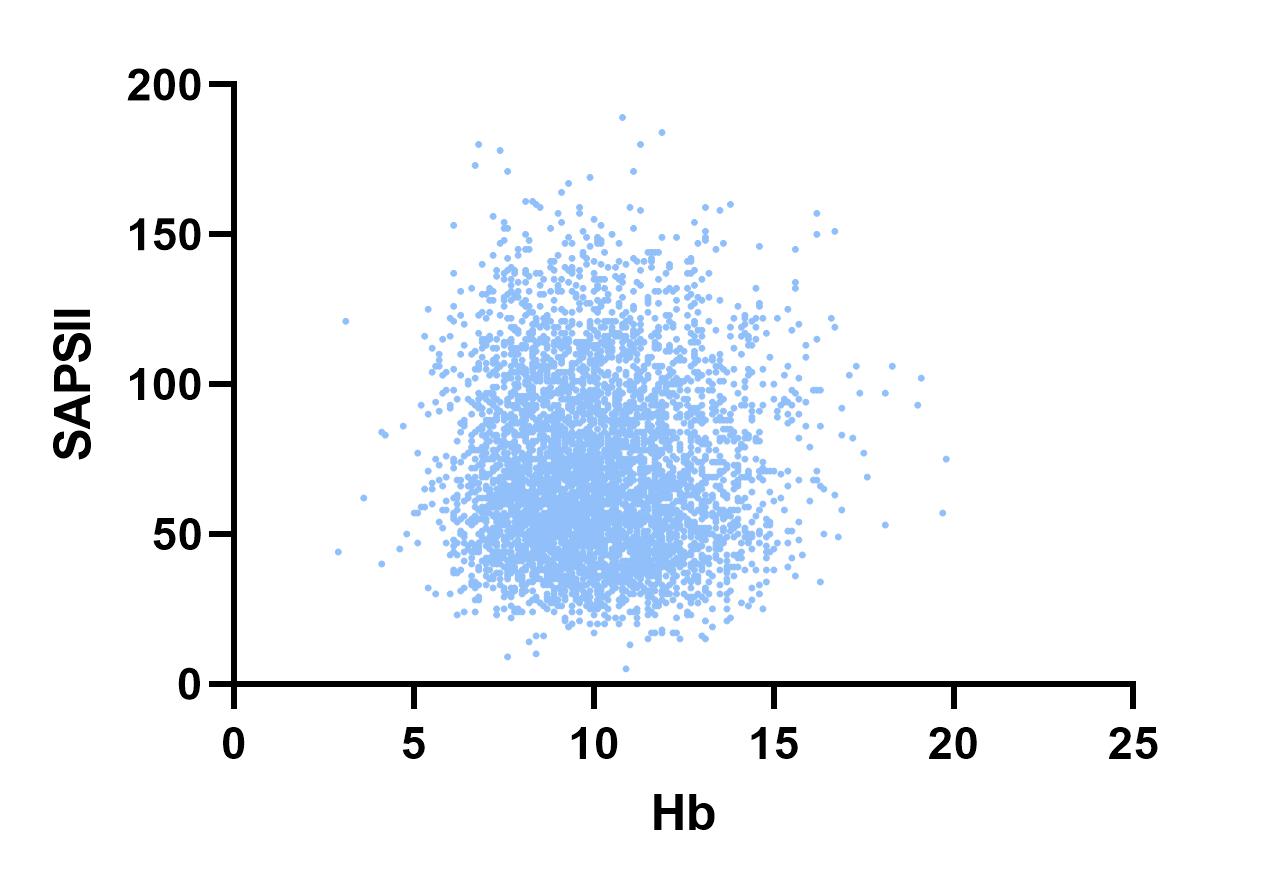

Supplement: S9 Fig — (JPG) [file pone.0313937.s012.jpg]
